# *ERCC1* and *ERCC2* Haplotype Modulates Induced BPDE-DNA Adducts in Primary Cultured Lymphocytes

**DOI:** 10.1371/journal.pone.0060006

**Published:** 2013-04-04

**Authors:** Xiaobo Lu, Yanhua Liu, Tao Yu, Sha Xiao, Xiaoyan Bao, Liang Pan, Guolian Zhu, Yuan Cai, Qiufang Liu, Cuihong Jin, Jinghua Yang, Shengwen Wu, Li An, Tahar van der Straaten

**Affiliations:** 1 Department of Toxicology, School of Public Health, China Medical University, Shenyang, People’s Republic of China; 2 Department of Occupational and Environmental Health, School of Public Health, China Medical University, Shenyang, People’s Republic of China; 3 Department Clinical Pharmacy and Toxicology, Leiden University Medical Center, Leiden, The Netherlands; University of Texas Health Science Center at San Antonio, United States of America

## Abstract

**Background:**

Benzo[a]pyrene(B[a]P), and its ultimate metabolite Benzo[a]pyrene 7,8-diol 9,10-epoxide (BPDE), are classic DNA damaging carcinogens. DNA damage caused by BPDE is normally repaired by Nucleotide Excision Repair (NER), of which *ERCC1* and *ERCC2/XPD* exert an indispensable role. Genetic variations in *ERCC1* and *ERCC2* have been related to DNA repair efficiency. In this study we used lymphocytes from healthy individuals to show that polymorphisms in *ERCC1* and *ERCC2* are directly associated with decreased DNA repair efficiency.

**Methods:**

*ERCC1* (rs3212986 and rs11615) and *ERCC2* (rs13181, rs1799793 and rs238406) were genotyped in 818 healthy Han individuals from the northeast of China. BPDE induced DNA adducts in lymphocytes were assessed by high performance liquid chromatography (HPLC) in 282 randomly selected participants. The effect of *ERCC1* rs3212986 and *ERCC2* rs238406 on DNA damage caused by B[a]P was assessed with a modified comet assay.

**Results:**

We found that the variant genotypes of *ERCC1* rs3212986 and *ERCC2* rs238406 were associated with the high levels of BPDE-DNA adducts. Especially *ERCC1* rs3212986 A-allele variant was significantly associated with the high BPDE-DNA adducts. Haplotype analysis showed that the *ERCC1* haplotype AC (OR = 2.36, 95% CI = 1.84–2.97), *ERCC2* haplotype AGA (OR = 1.51, 95% CI = 1.06–2.15) and haplotype block AGAAC (OR = 5.28, 95% CI = 2.95–9.43), AGCAC (OR = 1.35 95% CI = 1.13–1.60) were linked with high BPDE-DNA adducts. In addition, we found that the combined minor alleles of *ERCC1* rs3212986 and *ERCC2* rs238406 were associated with a reduced DNA repair capacity.

**Conclusions:**

Our results suggest that the variant genotypes of *ERCC1* rs3212986 and *ERCC2* rs238406 are associated with decreased repair efficiency of BPDE induced DNA damage, and may be predictive for an individual’s DNA repair capacity in response to environmental carcinogens.

## Introduction

Benzo[a]pyrene(B[a]P) is a classic DNA damaging carcinogen which is one of a multitude of polycyclic aromatic hydrocarbons(PAHs) commonly found in tobacco smoke and in the ambient environment [1], [2]. Benzo[a]pyrene 7,8-diol 9,10-epoxide (BPDE), the ultimate metabolite of B[a]P, forms covalent BPDE-DNA adducts within a cell that damages the structure and function of biological macromolecules such as DNA and protein [3]. The covalent binding of reactive metabolites to DNA is suggested to be involved in cancer initiation [4]. Most species have developed adaptive DNA repair systems against genomic insults from environmental hazards, and have effectively maintained genomic integrity during the evolution. An efficient DNA repair system is crucial for eliminating BPDE-DNA adducts, and a reduced DNA repair efficiency is related to a higher risk of cancer development.

DNA repair is a complicated biological process consisting of several distinct pathways. There are at least 5 DNA repair systems known to repair DNA damages: Base Excision Repair (BER), Nucleotide Excision Repair (NER), Mismatch Repair (MMR), Homologous Recombination Repair (HRR), and Non-Homologous End Joining (NHEJ). Nucleotide Excision Repair (NER) is an important and versatile repair system that removes a wide variety of DNA damages and especially deals with bulky DNA damage that leads to a distortion of the DNA helix such as DNA adducts induced by chemical carcinogens [5]. NER consists of a multi-step process that involves at least 20–30 proteins in a well-defined order. *ERCC1* (Excision repair cross complementation group 1) and *ERCC2*/*XPD* (Excision repair cross complementation group 2/xeroderma pigmentosum D) are both indispensable genes for a well functional NER. *ERCC1* protein forms a heterodimer with *ERCC4*/*XPF* and acts as an endonuclease that excises the DNA lesion by 5′ incision [6]. The *ERCC2* gene product *XPD* acts as a subunit of the basal transcription factor TF2/TFIIH complex and is also essential for NER. It encodes an ATP-dependent DNA helicase and opens DNA strands around the site of the lesion to make it accessible for repair by other NER proteins [7], [8]. *ERCC1* and *ERCC2* genes are both located on chromosome 19q13.3 and exert important roles as a whole. Genetic variations in the form of single nucleotide polymorphisms (SNPs) in *ERCC1* and *ERCC2* may modulate the levels of DNA damage in response to carcinogen exposure because of a possibly altered protein function or gene expression.

Two common SNPs in *ERCC1*, the synonomous 11615 at exon 4 (Asn118Asn), and rs3212986 located at the 3′-untranslated region of *ERCC1* (C8092A) have been associated with an increase risk to develop lung cancer [9]–[12], squamous cell carcinoma of the head and neck (SCCHN) [13], [14], basal cell carcinoma (BCC) [15]–[17], breast cancer [18], [19] and colorectal cancer [20]. Several SNPs have been identified in *ERCC2* and three of them are explored in our current study. *ERCC2* rs13181 at exon23 and rs1799793 at exon10 result in amino acid change (Lys751Gln and Asp312Asn, respectively) while rs238406 at exon6 is a silent polymorphism (Arg156Arg). Studies on these three polymorphisms have been reported extensively for their potential implication in the risk of cancer development. These three *ERCC2* SNPs are found to be associated with a reduced repair of aromatic DNA adducts [21], [22] and an increasing risk of lung cancer [23], [24], bladder cancer [25], esophageal squamous cell carcinoma (ESCC) [26] and head and neck cancer [27]. Although many population-based case-control studies suggested that these polymorphisms may predict an individual’s susceptibility to cancer, these conclusions are not entirely consistent and it is therefore imperative to investigate whether these genetic variations correlates with differences in DNA repair efficiency in the general population.

BPDE-DNA adducts detected in peripheral blood lymphocytes, are phenotypic markers for carcinogen metabolism and host DNA repair capacity [28]. However, the levels of in vivo-induced DNA adducts depend on the dose and duration of carcinogen exposure and are hardly estimated in a population-based study. For that reason we performed an in vitro study by incubating freshly isolated lymphocytes with BPDE to induce DNA adducts and determined the amount of adducts after standardized exposure conditions to indicate individual’s DNA repair capacity as the previous studies recommended [28], [29]. Because the amount of in vitro BPDE-induced DNA adduct levels are more than1000 times higher than those in vivo induced by smoking or other environmental carcinogens [30], measuring the detectable level of in vitro BPDE-induced DNA adducts in peripheral blood lymphocytes may provide a useful tool for measuring DNA repair capacity, which can reflect individual susceptibility to PAHs-induced carcinogenesis.

We hypothesize that *ERCC1* and *ERCC2* polymorphisms may modulate the induced BPDE-DNA adduct levels and affect host DNA repair capacity. Therefore, we tested DNA repair efficiency in lymphocytes from 282 individuals and associated that with their corresponding *ERCC1* and *ERCC2* genotypes. We found that the combined minor alleles of *ERCC1* rs3212986 and *ERCC2* rs238406 show a decreased DNA repair efficiency and can be used as valid biomarkers to predict an individual’s DNA repair capacity in response to environmental carcinogen.

## Materials and Methods

### Study Population

The study population consisted of 818 healthy participants recruited from a Physical Examination Center of Shenyang (A city in the northeast of China) from October 2010 to July 2011. The Institutional Review Board of China Medical University approved the study and informed consent was obtained from all participants prior to the study. All activities involving human subjects were done under full compliance with government policies and the Helsinki Declaration. After the study procedures were explained and all questions were answered, subjects signed informed consent forms. Demographic data (demographic characteristics, anamnesis, family history and lifestyles: smoke and alcohol consumption) were obtained with a questionnaire.

### DNA Isolation and Genotyping

From each participant 10 ml of venous blood was obtained by vena puncture and collected in tubes containing folic acid sodium anticoagulation. DNA was extracted from 2 ml blood by phenol chloroform extraction which has been described elsewhere [31]. Two SNPs in *ERCC1* (rs11615 and rs3212986) and three SNPs in *ERCC2* (rs13181, rs1799793 and rs238406) were analyzed by TaqMan® on ABI 7500 Real-time PCR system (ABI, US, Stagapore). All PCR reagents were purchased from ABI Company. Assay ID of *ERCC2/XPD* rs238406 is C_8714009_10 and the part number is 4351375.

*ERCC1* rs11615 primers:

Forward: 5′-CCTTCGTCCCTCCCAGA-3;

Reverse: 5′-CCCAGCACATAGTCGGGAAT-3′.

Probes: 5′-FAM-CGTGCGCAA**C**GTGCCCTG-MGB-3′;

5′-VIC-TCGTGCGCAA**T**GTGCCCTG-MGB-3′.

*ERCC1* rs3212986: primers:

Forward : 5′-GCTTTCTTTAGTTCCTCAGTTTCCC-3′;

Reverse: 5′-CAG TGC CCC AAG AGG AGA TG-3′.

Probes: 5′-FAM-TGC TGC TGC T**G**C TTC CGC TTC-MGB-3′;

5′-VIC-CTG CTG CTG CT**T** CTT CCG CTT CTT-MGB-3′.

*ERCC2/XPD* rs13181 primers:

Forward: 5′-CAG GAG TCA CCA GGA ACC GT NFQ-3′.

Reverse: 5′-CTC AGC CTG GAG CAG CTA GAA T-3′.

Probes: 5′-FAM-ATC CTC T**T**C AGC GTC T-MGB-3′.

5′-VIC-TCC TCT **G**CA GCG TC-MGB NFQ-3′.

*ERCC2/XPD* rs1799793 primers:

Forward: 5′-CCGCAGGATCAAAG AGACAGA-3′.

Reverse: 5′-CCTCTGCGAGGAGACGCTAT-3′.

Probes: 5′-FAM-CCG TGC TGC CC**G**_ACG AAGT-MGBNFQ-3′.

5′-VIC-CGT GCT GCC C**A**A CGA AGT GC-MGB NFQ-3′.

PCR reactions were run in a 20 µl final volume including: Premix Ex Taq^TM^10.0 µl, 0.4 µl of each probe and primer, 2 µl DNA (l0 ng/ul) and 6.4 µl pure water. Cycling conditions were 95°C for10 min, and 40 cycles of 95°C for 5s and 60°C for 34 s. Data analysis for allele discrimination was performed using SDS software.

### In vitro Lymphocytes Exposure to BPDE

Lymphocytes were isolated from 8 ml blood sample. In brief, blood was added gently above human lymphocyte separation medium in 15 ml centrifuge tubes. After centrifugation at 2000 rpm for 5 min, lymphocytes were separated and 10^7^cells per ml was suspended in frozen stock solution (containing 50% fetal bovine serum, 40% RPMI-1640 and 10% DMSO) and kept at −80°C promptly. Lymphocytes were re-suspended in RPMI l640 (supplemented with 20% fetal bovine serum,2 mM L-glutamine, 100 U penicillin streptomycin combination and 112.4 µg/ml phytohemagglutinin) at a cell content of 3×l0^5^/ml and were cultured for 2–3 days (logarithmic phase) in a 12-well cell culture plate under 5% CO_2_ at 3°C. After 67 hrs of phytohemagglutinin stimulation, a final concentration of 4 µmol/l BPDE (Benzo[a]pyrene-7,8-dihydrodiol-9,10-epocide, purchased from National Cancer Institute Chemical Carcinogen Repository, Midwest Research Institute, Kansas City, MO) was added to the cultures [28], [32]. The levels of BPDE-DNA adducts were maximal after 3 hrs incubation and decreased rapidly during 4–8 hrs [33], [34]. Therefore, lymphocytes were harvested after 5 hrs of incubation.

### Detection of BPDE-DNA Adducts Using High Performance Liquid Chromatography

**Figure 1:**
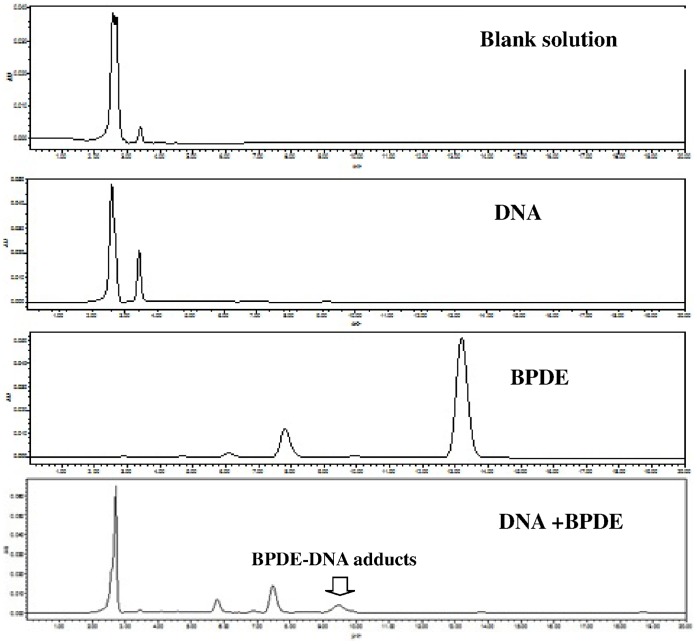
Chromatograms of blank, DNA, BPDE and DNA+BPDE solutions using HPLC-UV detection. Figure 1 reflects the representive HPLC readouts. We used the Chromatograms to detect BPDE-DNA adducts level. The retention time of 9.38 minute was identified to be BPDE-DNA adducts.

The harvested lymphocytes were washed and re-suspended in PBS. The HPLC methods used for detecting BPDE-DNA adducts was previously described [35], [36]. Briefly, DNA was extracted fro lymphocytes and quantified. The ultraviolet spectrum of DNA from calf thymus (obtained from the commercial source of Life Technologies Corporation) [37], PBS, BPDE standard solution and their mixture were scanned with a UV spectrophotometer to inspect the UV wavelength of the maximum absorption. The BPDE-DNA adducts were detected by a HPLC system using Waters Alliance 2487 with ultraviolet-absorbance detection at 275 nm. BPDE-DNA adducts were separated on the 250×4.6 mm Diamonsil(R) C_18_ 5 µm HPLC column (Dikma, Lake Forest, CA, USA) and were equilibrated in 70% methanol blended with 30% ultrapure water at a flow rate of 1 ml/min on column temperature of 30°C. 20 µl processed sample was injected into Rhoedyne and was analyzed for 20 min. The retention time of 9.38 minute was identified to be BPDE-DNA adducts by mass spectrometry (MS) in preliminary test. **Figure 1** shows the representative figures for HPLC readouts. Calibration was carried out with DNA from calf thymus and spiked with 0.01560,0.03250,0.06205,0.1250,0.2500,0.5000,1.000,2.000 µg/ml BPDE. The equation of linear regression (Y = 1068.37+3270.26X, R^2^ = 0.9856) was determined. The excellent accuracy, stability and recovery were confirmed by corresponding tests. The samples were then treated in the same way as these standard solutions. Quantification of BPDE-DNA adducts was determined by the peak-area of measurement using the linear regression curve for standard solutions expressed as fmol BPDE equivalents per microgram nucleotide. (1 fmol/µg DNA = 30 adducts/10^8^ nucleotides) [38].

### Detection of DNA Damage Caused by B[a]P Using the Modified Comet Assay

Anabiotic lymphocytes were incubated in 12-well plates overnight. Exponentially growing cells were divided into two groups evenly. One was incubated with 100 µg/ml B[a]P (Sigma Chemical Co., St. Louis, MO ) and S_9_ mixture for 5 hrs at 37°C, The other was incubated without B[a]P under the identical conditions. All cells were suspended in culture medium and centrifuged at 1500 r/min for 3 min at 4°C. Sediments were suspended in 0.5 ml cold PBS and 50 µmol H_2_O_2_ was added and kept on ice for 5 min. Pre-coated microscope slides were prepared by dropping 100 µl 1% NMA (normal melting point agarose dissolved in PBS, PH 7.4) to the glass slide and quickly cover it with a lid slide to make the gel evenly. After at least 10 minutes of incubation at 44°C, the lid slide was carefully removed. The 100 µl of cells and LMA gel mixture (80 µl cells and 300 µl of 1% low melting point agarose dissolved in PBS) was slowly added as a second layer, and incubated in dark at 4°C. After 10 min, 100 µl 1% LMA (low melting point agarose) was added as the last layer. Gel slides were soaked in cell lysis solution (100 mM disodium EDTA, 2.5 M NaCl, 10 Mm Tris-HCl pH 10.5, adding 1% Triton X-100 before using) for 60–90 min and immersed in the electrophoresis solution (1 M disodium EDTA, 300 mM NaOH, pH>13) for 30 minutes to denature DNA. After electrophoresis at 20 V (100 mA for 20 min), glasses were rinsed three times with 1 ml of neutralizing solution (0.4 M Tris-HCl, pH 7.5). Ethidium bromide was used for staining. Finally, DNA damage in a single cell was detected using fluorescent microscope. Mean difference values of tail olives moment (and tail areas) between control and B[a]P exposed group indicates presence of induced DNA damage of BPDE-DNA adducts. At least 30 tail olives moment (and tail areas) in each slide were counted. The damage level was obtained with the formula:

(control group-exposed group) tail olives moment (or tail area)/tail olives moment (or tail area) of control group.

**Figure 2:**
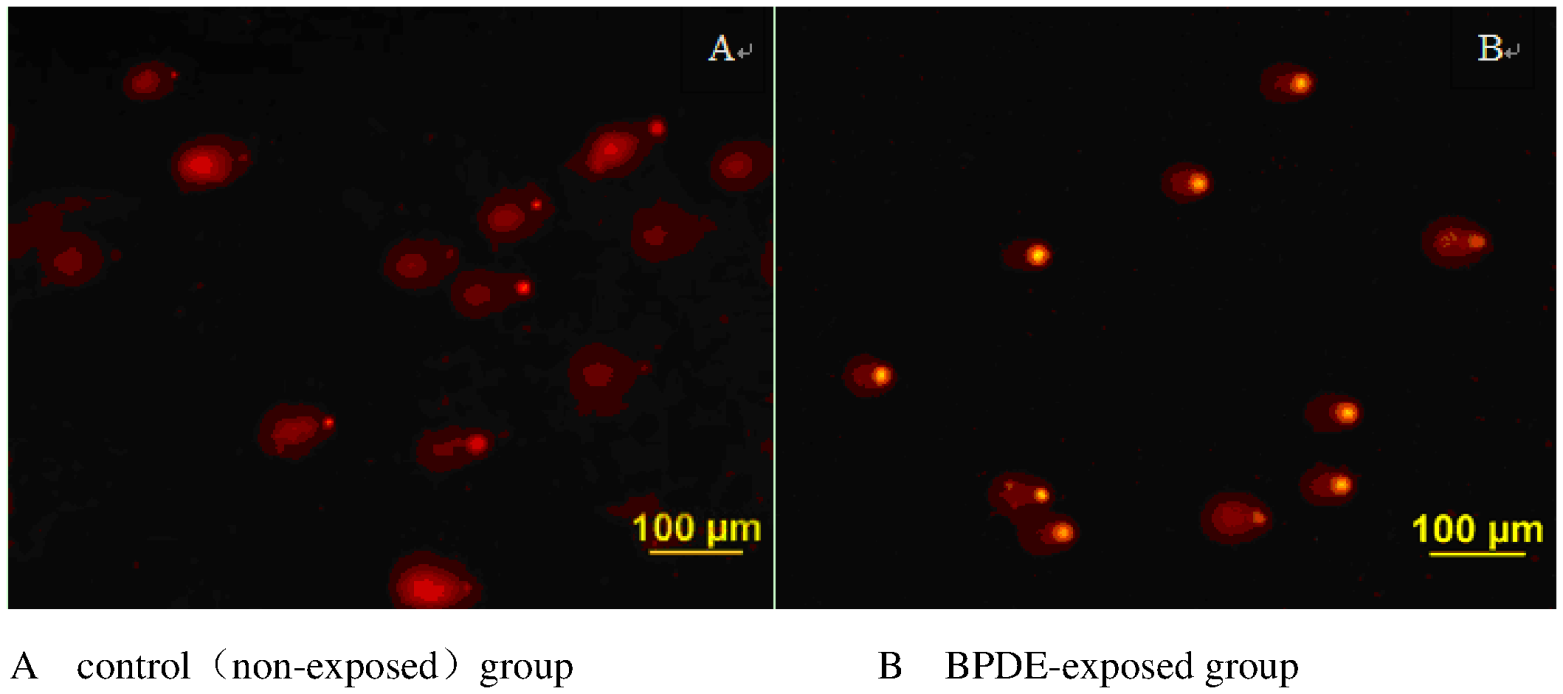
DNA damage caused by B[a]P exposure detected by a modified comet assay. Figure 2 shows a typical image to reflect the damage levels caused by BPDE-DNA adducts in a randomly selected sample. BPDE covalently binds to cellular DNA and forms interactive complexes. 50 µmol H_2_O_2_ was used to induce DNA fragmentation, resulting in long tails after electrophoreses in control lymphocytes (see Fig. 2A. control or non-exposed group). BPDE-DNA adducts will capture the short H_2_O_2_ induced DNA fragments, and consequently, a shorter tail olive (or tail area) will be found in BPDE-exposed cells compared to non-exposed cells (see Fig. 2B. BPDE-exposed group).

Mean ranks of tail olives moment and tail area ratio were denoted as holistic marking to reflect the integrated evaluation of DNA damage. A typical image of the modified comet assay used for data analysis has shown in **Figure 2**.

### Statistical Analysis

The Shapiro–Wilk normality test was used to test the normality of the DNA adducts distribution. According to the approximately normal distribution of BPDE-DNA adduct levels they were expressed as means±SD (standard deviation). The Hardy–Weinberg equilibrium was tested by a goodness-of-fit χ2 test to compare the observed genotype frequencies with the expected genotype frequencies, assuming the study population was in the Hardy–Weinberg equilibrium. The levels of linkage disequilibrium (LD) were analyzed by Haploview Software (version 4.1, Broad Institute). D′ shows the level of LD; R^2^ is the correlation coefficient between the two loci.

One-way ANOVA, non-parametric Kruskal Wallis tests and Wilcoxon two-sample test were used to determine DNA adduct levels by the categorical variables such as age, sex, smoking, drinking status and diverse genotypes. Multiple unconditional logistic regression analysis to estimate the odds ratios (ORs) and 95% confidence intervals (CIs) for high DNA adduct levels in participants with different haplotypes of *ERCC1* and *ERCC2*. All statistical analysis were two sided with *P* value <0.05 considered statistically significant and were performed using SPSS Software for Windows (version 15.0).

## Results

### 1. Distribution of *ERCC*1 and *ERCC2* Polymorphisms

A total of 818 healthy Chinese Han participants were included in the study. 238 were women and 580 were men; ages ranged from 17 to 90 years (mean 43 years). Genotype frequencies of *ERCC1* and *ERCC2* polymorphisms are described as following. For *ERCC1* rs11615, the frequency of C-allele was 0.729 and T-allele was 0.271, for rs3212986 the frequency of C-allele was 0.710 and A-allele was 0.290. Similarly, for *ERCC2* rs13181, the frequency of A-allele was 0.922 and C-allele 0.078; for rs1799793 the frequency of G-allele was 0.929 and A-allele was 0.071; and for rs238406 the frequency of C-allele was 0.559 and A-allele 0.441. The five SNPs were all in Hardy-Weinberg equilibrium. Linkage disequilibrium was found in *ERCC1* rs11615 and rs3212986 (D’ = 0.859, R^2^ = 0.091); *ERCC2* rs1799793 and rs238406 (D’ = 1.0, R^2^ = 0.055); *ERCC2* rs13181 and rs1799793 (D’ = 0.498, R^2^ = 0.24); *ERCC2* rs13181 and rs238406 (D’ = 0.834, R^2^ = 0.037).

### 2. BPDE-DNA Adducts and Characteristics of Study Population

For a panel of 282 randomly selected samples the induced BPDE-DNA adducts in lymphocytes were detected by HPLC. The levels of in vitro DNA adducts ranged from 438 to 8646 adducts/10^8^ nucleotides. The associations of BPDE-DNA adducts with characteristics of study population are represented in **Table 1**. Subjects with an age of 50–70, and over 70 years old had higher BPDE-DNA adduct levels compared with participants younger than 30 years old. The amount of BPDE-DNA adducts was significantly increased (*P*<0.05) for participants with a history of alcohol use or smoking. There was no difference in BPDE-DNA adduct levels between male and female.

**Table 1:** BPDE-DNA adducts levels and characteristics of study population (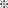, n = 282).

Covariates	N(%)	BPDE-DNA adduct levels(adducts/10^8^ nucleotides)	*P* a	*P* b
**Gender**				
Male	175(62.1)	2280.6±1310.8	Reference	
Female	107(37.9)	2516.0±1519.9	0.180	
**Age (years)**				
≤30	68(24.1)	1970.6±995.1	Reference	
30–50(obtain50)	43(15.2)	2226.7±1269.9	0.286	
50–70	80(28.4)	2432.5±1209.8	**0.025**	
≥70	91(32.3)	2451.4±1289.1	**0.018**	0.072
**Smoking history**				
never	142(51.4)	2161.0±1071.6	Reference	
<20 years	74(32.6)	2386.9±1601.0	0.225	
≥20 years	66(16.0)	2632.5±1516.7	**0.015**	0.047
**Drinking history**				
never	166(58.9)	2151.4±1069.1	Reference	
<20 years	85(30.1)	2476.4±1460.2	0.089	
≥20 years	31(11.0)	2672.0±1690.0	**0.015**	0.028

a *P* value was obtained using the LSD test or t-test analysis comparing with reference.

b *P* value was obtained using one-way ANVOA.

### 3. BPDE-DNA Adducts and *ERCC1*, *ERCC2* SNPs

The results for the association between BPDE-DNA adducts and ERCC1, ERCC2 genotypes are summarized in **Table 2**. The amount of BPDE-DNA adducts in subjects carrying *ERCC1* rs3212986 CA and AA genotypes was significantly higher compared to subjects with the CC genotype(*P*<0.01). Similarly, the amount of BPDE-DNA adducts in subjects with *ERCC2* rs238406 CA and AA genotypes was higher compared to subjects with the CC genotype. The amount of BPDE-DNA adducts showed a weak downward trend with the number of the A-allele of *ERCC2* rs1799793, but no statistical significance was observed. No difference of BPDE-DNA adducts in *ERCC1* rs11615 and *ERCC2* rs13181 polymorphisms were found.

**Table 2:** *ERCC1*, *ERCC2* genotypes and BPDE-DNA adducts levels (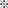, n = 282).

SNP	N(%)	BPDE-DNA adduct levers(adducts/10^8^ nucleotides)	*P* a	*P* b
***ERCC1*** ** C19007T, C>T, rs11615**				
CC	139(49.3)	2215.6±1216.7	Reference	
CT	113 (40.1)	2422.4±1052.0	0.151	0.306
TT	30 (10.6)	2187.0±1023.5	0.900	
CT+TT	143(50.7)	2373.0±1046.9	0.504	
***ERCC1*** ** C8092A, C>A, rs3212986**				
CC	162 (57.4)	1766.0±907.8	Reference	
CA	103 (36.5)	3140.1±1467.9	**<0.01**	**<0.01**
AA	17(6.1)	3458.7±1849.0	**<0.01**	
***XPD/ERCC2*** ** Lys751Gln, A>C, rs13181**				
AA	240(85.1)	2351.4±1427.1	Reference	
AC	40 (14.2)	2506.4±1219.8	0.517	0.710
CC	2 (0.7)	1861.0±1175.2	0.622	
AC+CC	42(14.9)	2475.6±1211.8	0.623	
***XPD/ERCC2*** ** Asp312Asn, G>A, rs1799793**				
GG	241(85.5)	2402.3±1440.4	Reference	
GA	39 (13.8)	2239.6±1086.7	0.500	0.306
AA	2 (0.7)	1006.5±286.4	0.160	
GA+AA	41(14.5)	2179.5±1093.7	0.141	
***XPD/ERCC2*** ** Arg156Arg, C>A, rs238406**				
CC	95(33.69)	2100.0±1164.1	Reference	
CA	140(49.64)	2350.1±1352.1	0.155	**0.003**
AA	47(16.67)	2944.9±1766.4	**0.001**	
CA+AA	187(66.31)	2507.1±1484.1	**0.041**	

a *P* value was obtained using the LSD test or t-test analysis comparing with reference.

b *P* value was obtained using one-way ANVOA.

### 4. Association of BPDE-DNA Adducts with *ERCC1* rs3212986 and *ERCC2* rs238406 Stratified by Age and Smoking Status

Previous studies have suggested that the levels of DNA adducts is affected by age and smoking status. **Table 3** **and** **Table 4** show the result of the association between *ERCC1* rs3212986, *ERCC2* rs238406 and BPDE-DNA adduct levels stratified by age and smoking status. *ERCC1* rs3212986 CA and AA genotypes were associated with high levels of BPDE-DNA adducts in each age group. For non-smokers and heavy smokers the minor A-allele of *ERCC1* rs3212986 was also found to be linked with the risk of high levels of BPDE- adducts. The minor A allele of *ERCC2* rs238406 was associated with high BPDE-DNA adduct levels in the age group above 70 years and in the group of heavy smokers.

**Table 3:** Association between *ERCC1* rs3212986 and *ERCC2* rs238406 polymorphisms and BPDE-DNA adducts stratified by age.

		≤30 years		OR(95% CI)	30–50 years (50 years)		OR(95% CI)
	<2055.5		≥2055.5		<2055.5	≥2055.5	
***ERCC1*** ** C8092A, C>A, rs3212986**							
CC	30		8	Reference	20	7	Reference
CA+AA	8		23	**10.78(3.52–33.06)**	2	14	**20.00(3.61–110.96)**
***XPD/ERCC2*** ** Arg156Arg, C>A, rs238406**							
CC	12		7	Reference	13	8	Reference
CA	22		17	1.33(0.43–4.09)	6	7	1.90(0.47–7.70)
AA	3		7	4.0(0.77–20.65)	3	6	3.25(0.63–16.79)
CA+AA	25		24	1.65(0.56–4.88)	9	13	2.35(0.69–7.98)
	**50–70 years (70years)**			**OR(95% CI)**	**>70 years**		**OR(95% CI)**
	**<2055.5**		**≥2055.5**		**<2055.5**	**≥2055.5**	
***ERCC1*** ** C8092A, C>A, rs3212986**							
CC	33		17	Reference	33	15	Reference
CA+AA	5		39	**11.65(3.83–35.45)**	11	27	**5.40(2.13–13.68)**
***XPD/ERCC2*** ** Arg156Arg, C>A, rs238406**							
CC	14		14	Reference	18	9	Reference
CA	19		22	1.58(0.42–3.03)	22	25	2.27(0.85–6.08)
AA	5		6	1.20(0.30–4.86)	6	11	**3.67(1.02–13.14)**
CA+AA	24		28	1.17(0.47–2.93)	28	36	**2.57(1.00–6.59)**

**Table 4:** Association between *ERCC1* rs3212986 and *ERCC2* rs238406 polymorphism and BPDE-DNA adducts stratified by smoking index.

	Smoking index (1)		OR (95% CI)	Smoking index (1–500)		OR (95% CI)	Smoking index (>500)		OR (95% CI)
	<2055.5	≥2055.5		<2055.5	≥2055.5		<2055.5	≥2055.5	
***ERCC1*** ** C8092A, C>A, rs3212986**									
CC	57	23	Reference	40	13	Reference	18	11	Reference
CA	15	41	**6.78(3.15–14.55)**	6	28	14.36(4.87–42.33)	1	12	**19.64(2.23–172.59)**
AA	2^ 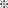 ^	7	**8.67(1.68–44.91)**	2^?^	6	**9.23(1.66–51.46)**	–	–	–
***XPD/ERCC2*** ** Arg156Arg, C>A, rs238406**									
CC	25	17	Reference	12	13	Reference	18	10	Reference
CA	32	33	1.51(0.69–3.33)	18	28	1.44(0.54–3.84)	8	21	**4.73(1.54–14.52)**
AA	13	22	2.49(0.99–6.26)	1	2	–	3	6	3.60(0.74–17.60)
CA+AA	45	55	1.80(0.87–3.74)	19	30	1.46(0.55–3.86)	11	27	**4.42(1.56–12.55)**

Smoking index 1: never smoking;

Smoking index = average cigarette numbers/day×years.

The interactive effect of all covariates on BPDE-DNA adduct levels was analyzed by Multiple Linear Regression according to the approximately normal distribution of BPDE-DNA adduct levels (**Table 5**). *ERCC1* rs3212986 and age had significant contribution to the variation of BPDE-DNA adduct levels among all covariates (*P*<0.05). The standardized partial regression coefficients obtained from Stepwise Multiple Linear Regression analysis can show the contribution to the variation of BPDE-DNA adduct levels. The coefficients of *ERCC1* rs3212986 and age were 0.470 and 0.149 respectively (*P*<0.05).

**Table 5:** Multiple covariates analysis for BPDE-DNA adduct.

Covariates	*β_j_*	*β_s_*	*P* d
*ERCC1* C8092A,C>A, rs3212986	910.742	**0.460**	**0.000**
*XPD/ERCC2* Arg156Arg, C>A, rs238406	44.435	0.025	0.657
Age	157.402	**0.149**	**0.010**
Smoking history	169.946	0.097	0.151
Drinking history	−91.796	−0.056	0.386

*β_j_* partial regression coefficient of multiple linear regression.

*β_s_* standardization partial regression coefficient of multiple linear regression.

d *P* value for the partial regression coefficient of all covariates to BPDE-DNA adduct levels using multiple linear regression.

### 5. BPDE-DNA Adducts and *ERCC1*, *ERCC2* Haplotypes

**Table 6** shows the association between BPDE-DNA adduct levels and *ERCC1* and *ERCC2* haplotypes. Median DNA adduct level was used as the cut off point to dichotomize the study participants. As a result, DNA adducts less than 2055.5 were categorized as low-level adduct groups, and more or equal to 2055.5 were categorized as high-level adduct groups. The participants with the most common haplotype showed the lowest risk for high BPDE-DNA adduct levels (**Table 2**). The haplotype including the minor A allele of *ERCC1* rs3212986 had a higher risk of high amounts of BPDE-DNA adducts. Compared with the reference, high BPDE-DNA adduct levels were associated with *ERCC1* haplotype AC (OR = 2.36, 95% CI = 1.84–2.97). For the most common *ERCC2* haplotype AGC, the frequency was 0.480. Compared with AGC, high BPDE-DNA adduct levels were associated with AGA haplotype which included the minor A-allele of *ERCC2* rs238406 (OR = 1.51, 95% CI = 1.06–2.15). Haplotype blocks combining the five SNPs in *ERCC1* and *ERCC2* are listed in **Table 5** **.** Six haplotype blocks had frequencies of >3% in the study population. Haplotype AGCCC is the most common one and is composed of the low-risk alleles of the five SNPs. The frequency of the most common haplotype AGCCC was 0.283. Haplotype block AGAAC including both the minor A-allele of *ERCC1* rs3212986 and *ERCC2* rs238406 (OR = 5.28 95% CI = 2.95–9.43); haplotype block AGCAC including the minor A-allele of *ERCC1* rs3212986 (OR = 1.35 95% CI = 1.13–1.60) were significantly associated with the risk of high BPDE-DNA adduct levels.

**Table 6:** *ERCC1*, *ERCC2* haplotypes and BPDE-DNA adduct levels.

Haplotype	Frequency	BPDE-DNA adduct levels		OR (95% CI)	*P* c
		<2055.5, N(%)	≥2055.5, N(%)		
***ERCC1*** ** C8092A C>A, rs3212986; ** ***ERCC1*** ** C19007T C>T, rs11615**					
CC	0.464	162(57.45)	99(35.11)	Reference	<0.001
CT	0.293	90(31.91)	76(26.95)	1.38(0.93–2.05)	0.1997
AC	0.230	30(10.64)	100(35.46)	**2.36(1.84–2.97)**	<0.001
AT	0.013	0(0)	7(2.48)		0.0118
***XPD/ERCC2*** ** Lys751Gln A>C, rs13181; ** ***XPD/ERCC2*** ** Asp312Asn G>A, rs1799793; ** ***XPD/ERCC2*** ** Arg156Arg C>A, rs238406**					
AGC	0.480	148(52.48)	123(44.62)	Reference	0.039
AGA	0.407	102(36.17)	128(45.39)	**1.51(1.06–2.15)**	0.030
CAC	0.041	11(3.90)	13(4.61)	1.02(0.44–2.35)	0.700
AAC	0.035	13(4.61)	7(2.48)	0.65(0.25–1.65)	0.118
CGC	0.029	8(2.84)	11(3.90)	1.65(0.65–4.24)	0.707
***XPD/ERCC2*** ** Lys751Gln A>C, rs13181; ** ***XPD/ERCC2*** ** Asp312Asn G>A, rs1799793; ** ***XPD/ERCC2*** **Arg156Arg C>A, rs238406; ** ***ERCC1*** ** C8092A C>A, rs3212986; ** ***ERCC1*** ** C19007T C>T, rs11615**					
AGCCC	0.283	97(34.40)	63(22.34)	Reference	0.001
AGACC	0.126	48(17.02)	23(8.16)	1.00(0.84–1.21)	0.002
AGCCT	0.136	43(15.25)	34(12.06)	1.10(0.84–1.45)	0.289
AGACT	0.11	33(11.70)	30(10.64)	1.09(0.94–1.26)	0.67
AGAAC	0.166	21(7.45)	72(25.53)	**5.28(2.95–9.43)**	<0.001
AGCAC	0.054	8(2.84)	23(8.16)	**1.35(1.13–1.60)**	0.004
Other^ 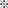 ^	0.125	32(11.34)	37(13.11)	**1.78(1.01–3.15)**	0.059

Other^
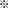
^ haplotypes with frequency less than 0.03.

c *P* value was obtained using X^2^ test.

### 6. The Combined Minor Alleles of *ERCC1* rs3212986 and *ERCC2* rs238406 Predict the DNA Damage Induced by B[a]P

**Figure 3:**
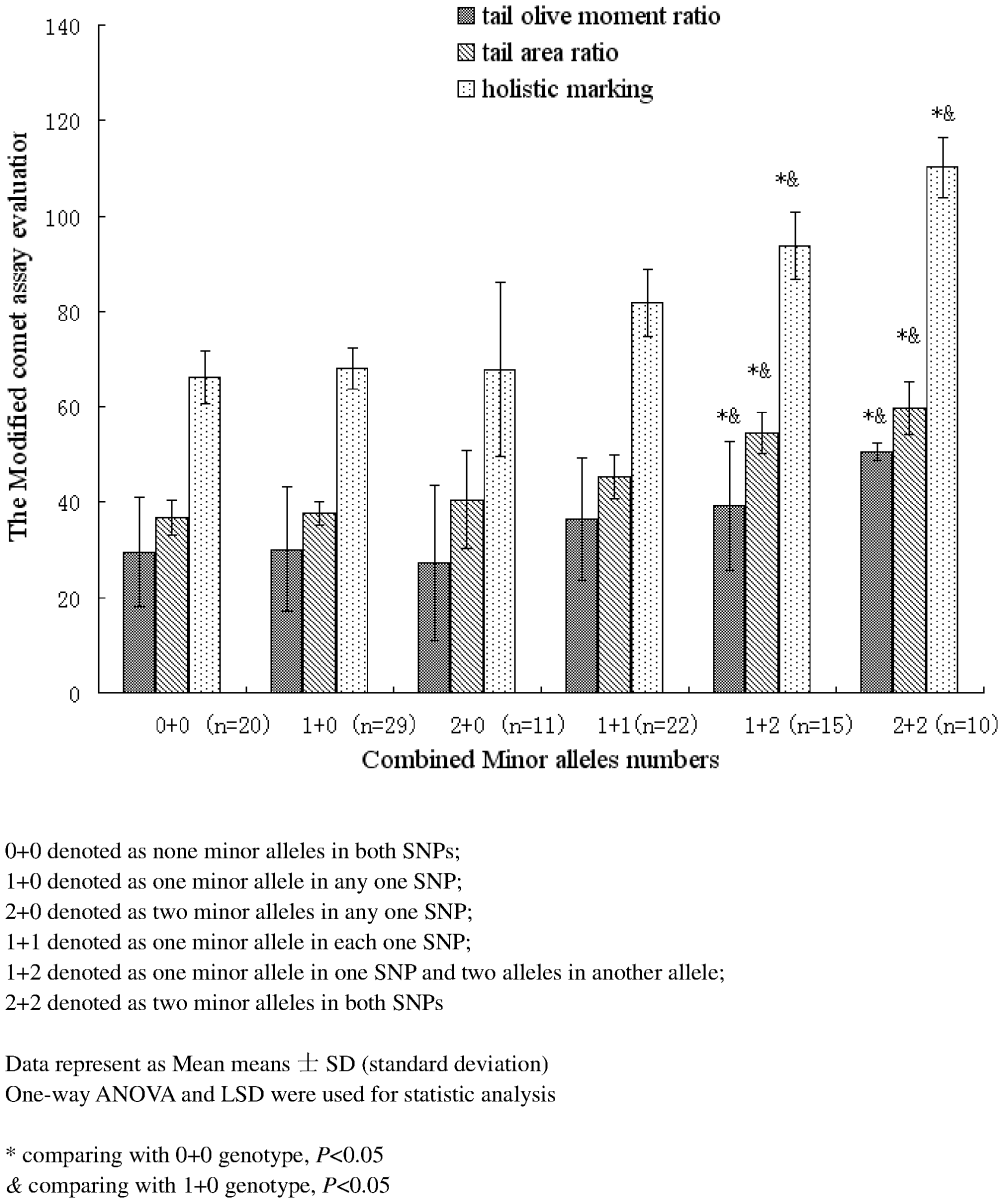
Comparison of DNA damage caused by B[a]P in different combined minor alleles of ERCC1 rs3212986 and ERCC2 rs238406. We selected the participants carrying different ERCC1 rs3212986 and ERCC2 rs238406 genotypes and analyzed their induced DNA damage levels induced by B[a]P using the modified comet assay. The damage levels were evaluated by the tail olive moment ratio, tail area ratio and the combined holistic marking respectively. The relationship between the combined minor alleles of ERCC1 rs3212986 and ERCC2 rs238406 and the effect on the repair efficacy of the DNA damage level caused by B[a]P were evaluated. Interestingly, we found following the increasing copies of the combined minor alleles, a reduced DNA repair capacity had been found in the tail olive moment ratio, tail area ratio and the combined holistic marking. (*P*<0.01).

Based on the results from the analysis of in-vitro BPDE-DNA adducts, we hypothesized that the minor alleles of *ERCC1* rs3212986 and *ERCC2* rs238406 are associated with reduced DNA repair capacity. To test our hypothesis, we selected the participants carrying different *ERCC1* rs3212986 and *ERCC2* rs238406 genotypes and analyzed their induced DNA damage levels by B[a]P using the modified comet assay. Interestingly, we observed that the combined minor alleles of *ERCC1* rs3212986 and *ERCC2* rs238406 were associated with a reduced DNA repair capacity by detecting differences in the tail olive moment ratio, tail area ratio and the comprehensive holistic marking (*P*<0.01)(See **Figure 3**).

## Discussion

In this study we investigated the effect of common SNPs in DNA repair enzymes *ERCC1* and *ERCC2* and their effect on repairing BPDE induced DNA damage. We found that haplotype of minor alleles of *ERCC1* rs3212986 and *ERCC2* rs238406 were associated with a reduced DNA repair capacity.

In recent years many studies have shown that reduced DNA repair efficiency is associated with an increased risk to develop cancer after exposure to environmental carcinogens. Hospital based case–control studies were usually performed in research fields, but some limitations in those studies such as selection bias and the retrospective nature of the study designs should be concerned. Therefore it is imperative to investigate the effect of genetic variations on DNA repair capacity in the general population. To our knowledge, only a few studies have investigated the associations between in vitro-induced DNA adduct levels and genetic variations in DNA repair genes in normal cells from healthy individuals. For that reason, we performed a cell culture-based assay for experimentally induction of BPDE-DNA adducts in isolated lymphocytes from a healthy population from the northeast of China. However, since the in vitro induced DNA adducts detected by HPLC as a surrogate measure of an individual’s DNA repair capacity may be less reliable, we also conducted a modified comet assay to further confirm the effect of those significant polymorphisms. Comparing with the previous analogue studies, the strength of our study is that we used a more reliable evaluation combining both the detection of BPDE-DNA adducts and the genetic damage in lymphocytes measured by the modified comet assay to reflect individual’s DNA repair capacity.

As is well known, the Nucleotide Excision Repair (NER) removes a wide variety of DNA adducts induced by chemical carcinogens PAHs [5]. *ERCC1* and *ERCC2* genes located on chromosome 19q13.3 both exert important roles in NER. *ERCC1* and *ERCC2* polymorphisms may affect NER function and consequently levels of DNA damage in response to carcinogen exposure. In the present study we investigated the effect of *ERCC1* and *ERCC2* polymorphisms on the repair efficacy of genetic damage induced by carcinogens. We found that the variant genotypes of *ERCC1* rs3212986 and *ERCC2* rs238406 are associated with the induced BPDE-DNA adduct levels after adjusting for age and smoking status. Furthermore, we conducted a haplotype analysis with these five SNPs. Although a number of studies showed that *ERCC1* rs11615 is a predictive biomarker for better survival in patients treated with platinum-based chemotherapy [6], [39]–[42], this effect may attribute to the strong linkage disequilibrium between *ERCC1* rs11615 and rs3212986 (D’ = 0.859, R^2^ = 0.091). We also found that *ERCC1* haplotype AC including the minor A-allele of *ERCC1* rs3212986, *ERCC2* haplotype AGA including the minor A-allele of *ERCC2* rs238406 and haplotype block AGAAC including both the minor A-allele of *ERCC1* rs3212986 and the minor A-allele of *ERCC2* rs238406 are associated with an increased risk of high DNA adduct levels. Therefore, the significant dose-response relationship between genetic damage levels induced by environmental carcinogens and the numbers of the combined minor alleles of *ERCC1* rs3212986 and *ERCC2* rs238406 indicated that these are valid predictive values for an individual’s DNA repair capacity.

*ERCC1* plays a central role in NER as a heterodimer endonuclease, which excises the DNA lesion by the 5′ incision. Interestingly, *ERCC1* rs3212986 (located at the 3′-nontranslated region of *ERCC1*) [20] is overlapping with the downstream adjacent gene designated *CAST* (previously reported as Anti-Sense ERCC1, ASE-1) [43]. It appeared that *ERCC1* rs3212986 polymorphism was also located in the coding region of *CAST* resulting in amino acid change [44]. *CAST* serves as a component of preformed T-cell receptor complexes and transduces signals upon T-cell receptor stimulation and represents a new signaling pathway via the CD3ε-containing TCR signaling module [45]. *ERCC1* rs3212986 polymorphism results in an amino acid change in *CAST* protein. *CAST* is possibly involved in the RNA polymerase I transcription complex [46]. Our present study suggests that the minor A allele of *ERCC1* rs3212986, associated with high levels of the induced BPDE-DNA adducts, decreased the individuals’ DNA repair capacity likely by modulating *CAST* protein’s function. However, the exact role of *CAST* related to cancer susceptibility or DNA repair has not been elucidated yet.

A second polymorphism in *ERCC2* rs238406 was also found to be associated with the genetic variation of DNA repair capacity in a univariate analysis but not in a multiple linear regression analysis. Although the SNP is a silent polymorphism and no change occurs in the amino acids, Manuguerra et al reported that *ERCC2* rs238406 AA and CA compared with CC was associated with an increased risk of skin cancer [47]. Consistently with their finding, we also found that the minor A allele of *ERCC2* rs238406 was associated with high BPDE-DNA adduct levels and low DNA repair capacity. These results were also in line with three studies on *ERCC2* rs238406 polymorphisms based on the Chinese population [48]–[50]. However, Lovatt et al [51] reported that those genotypes were associated with a low risk of skin cancer. Chang JS et al [52] also reported that the minor A allele of *ERCC2* rs238406 had no relationship with the risk of cancer development. In contrast, Zhao et al found that the participants with *ERCC2* rs238406 AA or CA had a lower risk of having high-level adducts compared with *ERCC2* rs238406 CC after adjustment for other covariates and also reminded that the inconsistent findings in above studies are due to the sample size [28]. In our current study, besides the detection of DNA adducts we also performed the modified comet assay to further confirm the significant effect of *ERCC2* rs238406 on DNA damage caused by B[a]P. *ERCC2* rs238406 was validated to have a significant effect on DNA repair capacity. However, the detailed mechanisms need to be clarified systematically.

In the present study, the participants carrying *ERCC2* rs1799793 AA and GA genotypes had lower BPDE-DNA adduct levels than those with wild-type GG genotype; however this difference did not reach the limit of statistical significance. There was no statistical difference between the amount of BPDE-DNA adducts and the minor alleles of *ERCC2* rs13181 or rs1799793, which was inconsistent with data reported by Matullo G et al [53] in Italian, and by Hui Zhao et al [28] in non-Hispanic white participants. However, it was consistent with data reported by Zhai et al [54] in Chinese people. In contrast, Vineis P et al [5], Zhang J et al [55], Spitz MR et al [56] and Mitra AK et al [57] reported that *ERCC2* rs13181 and rs1799793 minor alleles are risk factors to cancer development and associated with low DNA repair capacity.

These inconsistent findings might be related to different ethnicities. Donghui Li et al reported that ethnicity was a significant predictor of BPDE-DNA adducts levels [30]. The prevalence of the *ERCC1* rs11615 C alleles according to Hapmap data from NCBI is 0.36 and 0.24 respectively in Caucasians and China respectively. Our current study showed that the frequency of the T-allele was 0.271, which is much lower than the average frequency in a European population but similar to other studies in China. For *ERCC1* rs3212986, the frequency of the A-allele was 0.290 and concordant with frequency in Caucasians and China. In Caucasians, the reported frequency of *ERCC2* rs13181 C-allele varied from 0.35 to 0.41, and the reported *ERCC2* rs1799793 A-allele frequency also varied from 0.28 to 0.40 [23]. In contrast, the frequency of the variant C-allele of *ERCC2* rs13181 was 0.078 and the A-allele of *ERCC2* rs1799793 was 0.071, which are consistent with frequencies in China but markedly different from previous reports in Caucasians. For *ERCC2* rs238406, the frequency of A-allele was 0.427 and consistent with frequency in Caucasians [58]. Although an analogue study also analyzed the induced BPDE-DNA adducts in cultured primary lymphocytes from 707 healthy non-Hispanic participants, whether the same associations exist in other ethnic groups is still unknown. Therefore it was imperative for us to perform a different study based on a population from the northeast of China.

Overall, we found a significant dose-response relationship between genetic damage levels and the variant genotypes of *ERCC1* rs3212986 and *ERCC2* rs238406. This indicated that the variant genotypes provided a valid predictive value to individual’s DNA repair capacity in response to environmental carcinogens. However, the limitation of our current study is obvious. The induced in-vitro DNA adducts in lymphocytes were only detected in 282 study participants (a part of 818 study participants) and we randomly selected 107 participants carrying suitable genotypes and measured their genetic damages caused by B[a]P using the comet assay. Furthermore, this assay is based on a healthy population from the northeast of China which may not provide a complete risk profile of cancer susceptibility related to environmental carcinogen all over China, or even all over the world. Therefore our research should be considered part of a battery of complementary assays needed for cancer risk assessment.

### Conclusions

Our results suggested that the genotypes and haplotypes of *ERCC1* rs3212986 and *ERCC2* rs238406 modulate the efficacy of the DNA repair system. The variant alleles of *ERCC1* rs3212986 and *ERCC2* rs238406 were found associated with a reduced DNA repair capacity and may serve as predictive value to an individual’s DNA repair capacity in response to environmental carcinogens.
